# Treatment With Evinacumab Links a New Pathogenic Variant in the LPL Gene to Persistent Chylomicronemia

**DOI:** 10.1210/jendso/bvaf025

**Published:** 2025-02-14

**Authors:** Miriam Larouche, Poulabi Banerjee, Diane Brisson, Robert Pordy, Daniel Gaudet

**Affiliations:** Université de Montréal and ECOGENE-21, Chicoutimi, QC G7H 7K9, Canada; Regeneron Pharmaceuticals Inc., Tarrytown, NY 10591, USA; Université de Montréal and ECOGENE-21, Chicoutimi, QC G7H 7K9, Canada; Regeneron Pharmaceuticals Inc., Tarrytown, NY 10591, USA; Université de Montréal and ECOGENE-21, Chicoutimi, QC G7H 7K9, Canada

**Keywords:** chylomicronemia, ANGPTL3, evinacumab, lipoprotein lipase deficiency

## Abstract

**Background:**

Persistent chylomicronemia is caused by lipoprotein lipase deficiency (LPLD) or lack of lipoprotein lipase (LPL) bioavailability. This disorder is characterized by plasma triglyceride (TG) levels above 10 mmol/L, increased acute pancreatitis risk, and features of familial chylomicronemia syndrome (FCS). Evinacumab is an angiopoietin-like protein 3 (ANGPTL3) monoclonal antibody, and its efficacy in decreasing plasma TG levels depends on LPL bioavailability.

**Objective:**

To identify FCS-causing pathogenic variants in patients with persistent chylomicronemia treated with evinacumab.

**Methods:**

A phase II clinical trial was conducted with evinacumab in patients with severe hypertriglyceridemia. Plasma TG values were measured at baseline and every 2 weeks for 24 weeks. Three FCS patients homozygotes for a P234L pathogenic variant in the LPL gene (HoLPL P234L) known to be associated with low postheparin LPL activity (proven LPLD) participated in the study and were used as tracers. The genotype-specific efficacy of evinacumab to decrease TG levels in other participants was compared to that achieved in HoLPL P234L patients.

**Results:**

After 24 weeks of evinacumab treatment, TG levels decreased <20% in HoLPL P234L patients known to lack LPL. Similarly, a participant homozygote for a E282X variant in the exon 6 of the LPL gene that was suspected to be pathogenic due to its location did not respond to evinacumab (TG decreased <10% and remained >10 mmol/L).

**Conclusion:**

The efficacy of ANGPTL3 inhibitors in decreasing TG levels is LPL-dependent. Poor response to evinacumab supports the evidence that the E282X variant in the LPL gene is pathogenic and associated with persistent chylomicronemia (FCS phenotype).

Persistent chylomicronemia is a rare metabolic disorder characterized by an accumulation of chylomicrons in the bloodstream and lipoprotein lipase deficiency (LPLD). Affected patients have plasma triglycerides (TG) chronically over 10 mmol/L and lactescent plasma and are at high risk of acute pancreatitis and other morbidities such as hepatic steatosis or cardiovascular disease [1-3]. Familial chylomicronemia syndrome (FCS) identifies persistent chylomicronemia that is caused by a biallelic combination of pathogenic variants in the lipoprotein lipase (LPL) gene or in genes necessary to its function specifically lipase maturation factor 1, glycosylphosphatidylinositol anchored high-density lipoprotein binding protein 1 (GPIHBP1), apolipoprotein(APO)-C2 or -A5, involved in LPL maturation, anchoring to the endothelial wall or binding to chylomicrons, and very low density lipoprotein (VLDL) [4, 5]. The effect of variants on LPL lipolytic activity is not always documented although LPL functional analyses previously performed among biallelic carriers of pathogenic LPL gene variants such as P234L or G215E [6, 7] revealed very low postheparin LPL activity and LPLD. Such variants can thus be used as tracers to identify new potentially pathogenic variants or factors associated with FCS or persistent chylomicronemia [8].

Angiopoietin-like protein 3 (ANGPTL3) is a strong LPL inhibitor, and its overexpression is associated with reduced TG-rich lipoproteins catabolism leading to increased plasma TG levels [9]. Genetic studies have demonstrated that naturally occurring loss-of-function variants are associated with hypolipidemia, which has prompted the development of ANGPTL3 inhibitors [10-13]. Evinacumab is a monoclonal antibody targeting ANGPTL3 with the potential to cover a large spectrum of dyslipidemias from homozygous hypercholesterolemia to severe hypertriglyceridemia (sHTG) (Fig. 1A) [14-16]. ANGPTL3 inhibitors upregulate LPL, and their efficacy to decrease TG levels and clear TG-rich lipoproteins relies on LPL bioavailability (Fig. 1B). Treating sHTG patients with evinacumab might thus contribute to identifying new FCS-causing variants or to elucidating the functionality of variant of uncertain significance in patients with persistent chylomicronemia. In this study, we discuss the case of a patient homozygote for a variant in the exon 6 of the LPL gene presenting sHTG and chylomicronemia that persists despite treatment with evinacumab.

**Figure 1.**
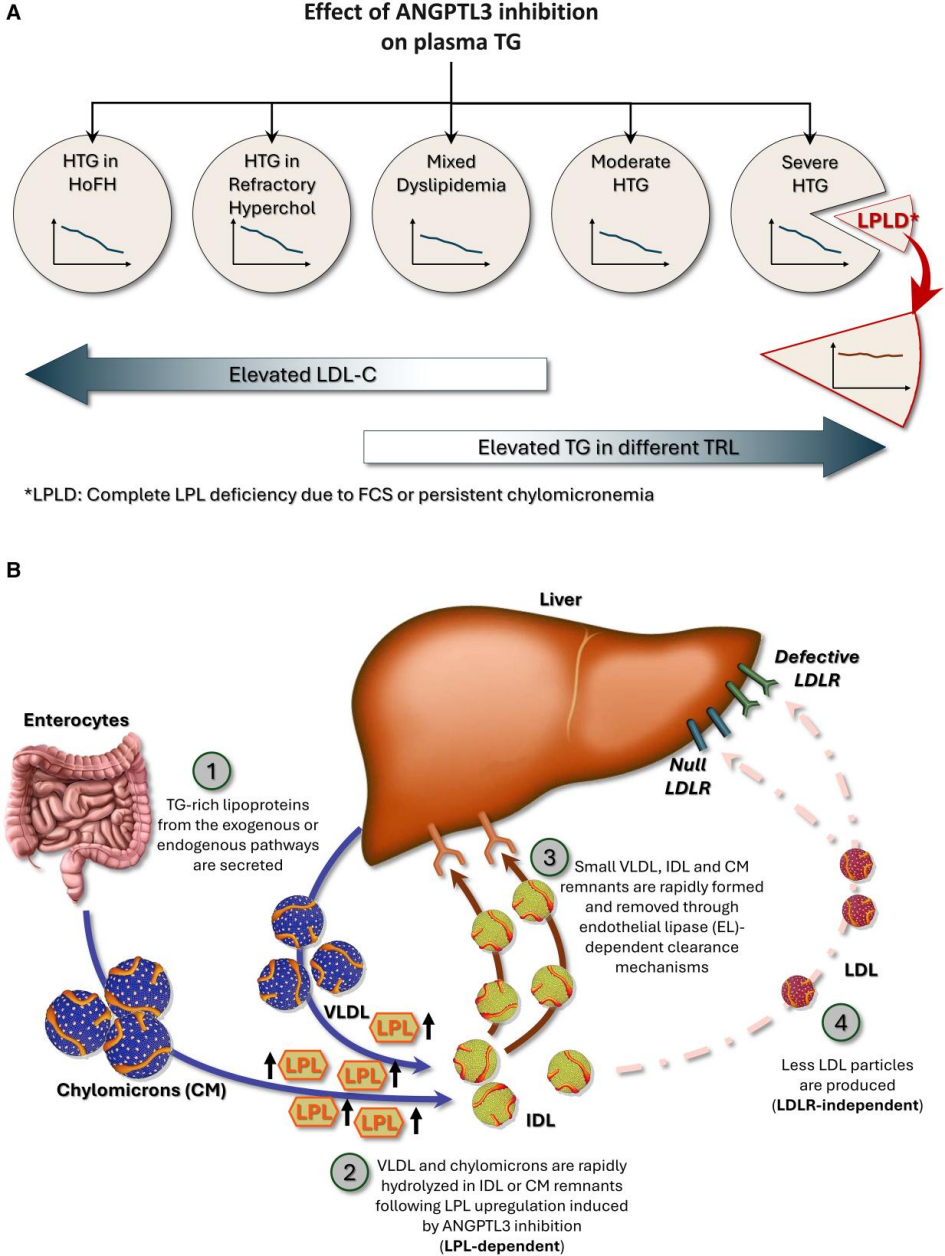
Spectrum and mechanisms of action of ANGPTL3 inhibition. ANGPTL3 inhibition decreases TG levels in a large spectrum of lipid disorders from homozygous familial hypercholesterolemia to persistent chylomicronemia provided that LPL is bioavailable (Fig. 1A). In patients with complete LPL deficiency and persistent chylomicronemia, ANGPTL3 inhibition does not decrease TG levels since it requires LPL to be effective (LPL-dependent). As illustrated in Fig. 1B, it is likely that following ANGPTL3 inhibition, TG-rich lipoproteins from both the exogenous or endogenous pathways (chylomicrons and VLDL) are secreted (1) and are rapidly hydrolyzed following LPL upregulation (2). IDL and chylomicrons remnants are then promptly removed from the bloodstream and internalized through endothelial lipase-dependent clearance mechanisms (3). Fewer LDL particles are formed, which allows evinacumab to be effective even in the absence of competent LDLRs (LDLR-independent pathway) (4). Abbreviations: ANGPTL3, angiopoietin-like protein 3; LPL, lipoprotein lipase; LDLR, lipoprotein lipase receptor; TG, triglycerides; VLDL, very low density lipoprotein.

## Method

A multicenter, phase 2, partially blind, placebo-controlled trial was conducted to evaluate the efficacy and safety of IV administration of evinacumab (RRID: AB_2911192) in patients with a history of sHTG (TG ≥ 10 mmol/L) or with baseline TG level above 5 mmol/L and a history of acute pancreatitis (NCT03452228) [16]. The study protocol was approved by institutional review boards (IRBs) and/or ethics committees (Quorum Review, Comitato Etico dell Universita, Policlinico Umberto I di Roma, North West—Greater Manchester South Research Ethics Committee, The University of Pennsylvania IRB, The University of Texas IRB, Western IRB, Human Research Protection Program, The University of Kansas Medical Center and Copernicus IRB). Details on the design and results of this study are reported elsewhere [16]. Briefly, the participants were divided in 3 cohorts. Cohort 1 comprised patients with chylomicronemia being monogenic or digenic homozygotes for pathogenic variants in the LPL, GPIHBP1, or APOC2 genes. Functional genotype-LPL activity correlations were not available in the majority of cases. There were, however, 3 homozygote for LPL pathogenic variants (HoLPL) P234L patients in this cohort that served as comparators of response to evinacumab to assess LPL bioavailability. Cohort 2 was composed of patients with multifactorial chylomicronemia who were heterozygote for LPL pathogenic variants whereas cohort 3 included chylomicronemic patients with other causes of sHTG or without genotype information. Each group received either IV evinacumab (15 mg/kg) or placebo in a monthly infusion for a period of 12 weeks double-blind, 12 weeks single-blind, and 12 weeks of follow-up. LPL activity was measured at baseline and at the end of treatment, and whole exome sequencing was done to determine or confirm the genotypes. Lipids and lipoproteins of which TG levels were measured every 2 weeks to assess efficacy and standard biochemistry, of which liver enzymes measurement, was done to assess the safety of the agent.

## Results

**Figure 2.**
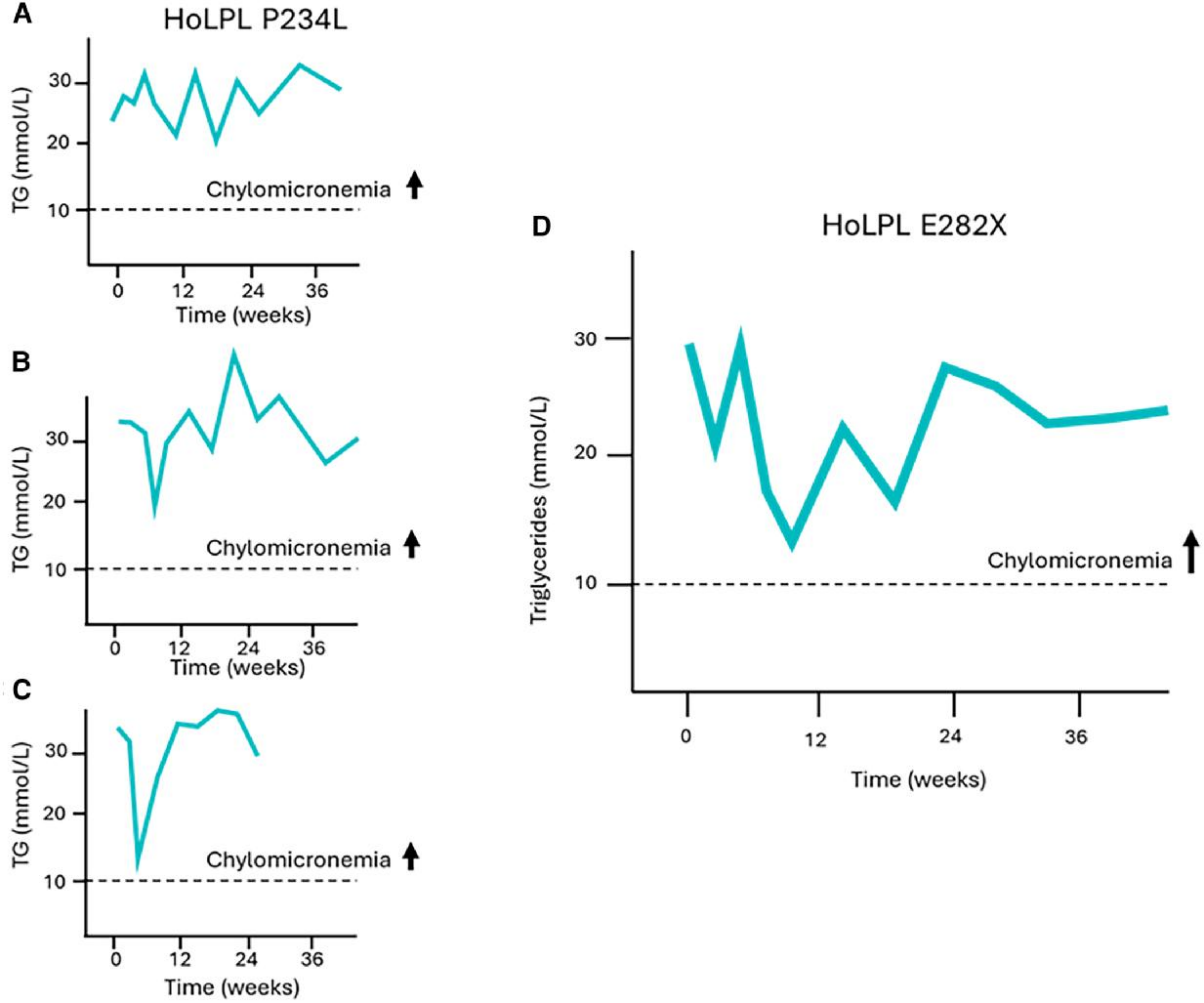
Exposure to evinacumab of 3 patients with proven LPL deficiency carrying known FCS-causing variants in the LPL gene and not responding to ANGPTL3 inhibition identified a new potentially pathogenic variant. In patients homozygotes for the P234L pathogenic variant (exon 5) completely lacking LPL activity due to defect at the catalytic site of the LPL protein, TG levels remain elevated and do not decrease below 10 mmol/L following evinacumab treatment (A, B, C). One homozygote carrier of the E282X variant did not respond either, and TG levels remained above 10 mmol/L (D). This variant is thus likely pathogenic and FCS-causing. It is located in exon 6 and surrounded by other documented variants associated with chylomicronemia. This region of the LPL gene codes for the enzyme catalytic site and affects lipoprotein binding. Abbreviations: ANGPTL3, angiopoietin-like protein 3; FCS, familial chylomicronemia syndrome; LPL, lipoprotein lipase; TG, triglycerides.

A total of 32 patients receiving the treatment and 15 on placebo completed the 12-week double-blind and 12-week single-blind periods. Baseline characteristics and detailed results of the study have been previously reported [16]. Three participants in cohort 1 were homozygotes for the P234L variant in the LPL gene. It has been demonstrated that this genotype is associated with FCS (persistent chylomicronemia) due to the absence or extremely low postheparin LPL activity [6, 7]. These 3 patients did not respond to evinacumab, and TG levels did not decrease after 24 weeks of treatment. Another participant was homozygote for the E282X variant in the LPL gene and responded poorly to evinacumab. This patient was a woman in her 50s who previously had 2 episodes of acute pancreatitis. She was not overweight (body mass index: 24.3 kg/m^2^), and despite treatment with fibrates and omega 3, her baseline TG level was >30 mmol/L. Following 24 weeks of treatment with evinacumab, TG levels decreased by less than 10% and never dropped below 10 mmol/L (Fig. 2), which characterizes chylomicronemia and is above the threshold of risk of acute pancreatitis. The response to evinacumab of this HoLPL E282X woman was thus similar to that of HoLPL P234L patients (Fig. 2).

## Discussion

Treatment with evinacumab, an agent that requires LPL to be effective, demonstrates that the E282X variant in the LPL gene is most likely pathogenic and associated with LPLD and persistent chylomicronemia. ANGPTL3 inhibitors directly target LPL upregulation (Fig. 1B). Although described as an anti-ANGPTL3 antibody, evinacumab was recently shown to be a potent inhibitor of the ANGPTL3/8 complex that is formed postprandially [17]. The ANGPTL3/8 complex is thought to be a significantly more potent circulating inhibitor of LPL than ANGPTL3 alone [18]. Patients lacking LPL poorly respond to evinacumab and ANGPTL3 or 3/8 complex inhibition as illustrated by the response observed in the 3 patients who were homozygotes for the known pathogenic and FCS-causing P234L variant, highlighting the LPL-dependent mechanism of action of evinacumab.

The cases studied in this manuscript were already considered FCS in a previous study and did not respond to evinacumab [16]. Poor response to ANGPTL3 inhibition could thus be an opportunity to better characterize the genetic architecture of patients with persistent chylomicronemia.

The P234L variant that we used as a tracer of LPLD is located on exon 5 of the LPL gene, whereas the E282X variant is located in exon 6 and induces a premature stop codon [19]. Known FCS-causing pathogenic variants are located in exon 6 of the LPL gene. In particular, the D277N and N318S are 2 missense variants that are located very close to the E282X variant. D277N is a pathogenic missense mutation resulting in a substitution of asparagine for aspartic acid. It is a null variant that inhibits the LPL catalytic activity and causes LPLD (FCS) in the homozygote state [20]. N318S is a missense variant leading to the substitution of an asparagine to serine. This variant is probably not pathogenic per se but most likely confers a susceptibility to sHTG by substantially decreasing LPL enzymatic activity [21]. This variant reduces the stability of the homodimer leading to decreased catalytic activity and higher TG levels [22, 23]. The E282X variant is located between these 2 documented variants and most likely impacts LPL catalytic activity leading to chylomicrons and VLDL accumulation. This variant is thus most likely pathogenic according to the guidelines for the interpretation of sequence variants issued by a joint consensus recommendation of the American College of Medical Genetics and Genomics [24]. Although most likely pathogenic, the E282X variant has not been previously associated with the FCS phenotype. In this study, the HoLPL E282X patient presented a history of chylomicronemia that persisted despite treatment with an agent such as evinacumab that requires LPL to be effective. It was associated with an FCS phenotype and a response to evinacumab that was similar to homozygotes for the LPL P234L pathogenic variant that is known to be FCS-causing and served as a tracer of LPL bioavailability. The pathogenicity of the LPL E282X variant was also supported by the obtention of high scores when using different FCS diagnosis scoring systems developed to accurately identify patients with persistent chylomicronemia and FCS [25-27]. Clinical scores fairly correlate with postheparin LPL activity [25]. Clinically, this patient had a prior history of recurrent acute pancreatitis, presented plasma TG sustainably >15 mmol/L, had a body mass index of 24.3 kg/m^2^, and did not respond to fibrates and omega 3.

Regardless of the percentage of variation in TG levels, what distinguishes persistent chylomicronemia from other sHTG phenotypes is that triglyceridemia sustainably remains above 10 mmol/L despite treatment. Chylomicronemia that persists following fibrate or omega-3 treatment is used as 1 of the criteria for defining FCS in most diagnosis scoring systems. Evinacumab is an inhibitor of ANGPTL3 (and 3/8 complex) that is importantly and specifically LPL-dependent and could thus be a more sensitive tool than the response to fibrate to assess LPL bioavailability and diagnose LPLD or FCS.

The poor response to evinacumab, the medical history, and the fact that plasma TG concentrations of this patient rarely decrease below 10 mmol/L despite fibrates or ANGPTL3 inhibition contribute to the evidence that the LPL E282X variant is pathogenic, FCS-causing, and most likely associated with LPLD just as the well-documented LPL P234L pathogenic variant.

## Data Availability

Restrictions apply to the availability of the data presented or analyzed during this study to preserve patient confidentiality. The corresponding author will on request detail the restrictions and any conditions under which access to some data may be provided.

## Trial Registration Number

ClinicalTrials.gov: NCT03452228

## Abbreviations

ANGPTL3: angiopoietin-like protein 3
ANGPTL8: angiopoietin-like protein 8
FCS: familial chylomicronemia syndrome
HoLPL: homozygote for lipoprotein lipase pathogenic variants
LPL: lipoprotein lipase
LPLD: lipoprotein lipase deficiency
sHTG: severe hypertriglyceridemia
TG: triglycerides
VLDL: very low density lipoprotein
